# Health psychology for more sustainable healthcare: a systems-oriented framework

**DOI:** 10.1080/21642850.2026.2745672

**Published:** 2026-10-10

**Authors:** Nicola Grignoli, Jeannette Büchel, Olivier Favre, Roger Keller, Myrta Isenschmid, Tiffany Martin, Serena Petrocchi, Chantal Martin Soelch, Jennifer Inauen

**Affiliations:** a Swiss Society for Health Psychology, Bern, Switzerland; b Cantonal Sociopsychiatric Organisation, Public Health Division, Department of Health and Social Care, Repubblica e Cantone Ticino, Mendrisio, Switzerland; c Institute for Family Medicine, Università della Svizzera italiana, Lugano, Switzerland; d Suva, Swiss National insurance Fund, Luzern, Switzerland; e Centre for Inclusion and Health in Schools, Zurich University of Teacher Education, Zürich, Switzerland; f Diabètevaud, (Vaud association for people living with diabetes), Lausanne, Switzerland; g Institute of Psychology, University of Fribourg, Fribourg, Switzerland; h Institute of Psychology, University of Bern, Bern, Switzerland

**Keywords:** Health psychology, integrated healthcare, health promotion, health behavior, healtcare systems

## Abstract

**Background:**

Healthcare systems face growing pressure from rising costs, workforce shortages, chronic disease burden, and increasing mental health needs. At the same time, many health outcomes depend on behavioural and psychosocial factors that remain insufficiently integrated into healthcare delivery. Although health psychology provides evidence-based approaches to prevention, behaviour change, and patient-centred care, its contribution often remains fragmented rather than structurally embedded within healthcare systems. This policy-oriented conceptual perspective aims to fill this gap and inform stakeholders about how the discipline can contribute to more sustainable healthcare systems through the integration of behavioural and psychosocial expertise.

**Method:**

This perspective and framework was developed through iterative discussions among the authors, experts from health psychology academia and practice, informed by established health psychology theories and selected interdisciplinary literature, with an emphasis on systematic reviews, large-scale studies, and authoritative policy sources.

**Results:**

We identified three interrelated domains of contribution of health psychology: (1) research, education, and policy support; (2) health promotion and prevention in schools, workplaces, and communities; and (3) interprofessional clinical integration in hospitals and primary care.

**Conclusions:**

Coordinated psychosocial integration across prevention and care pathways may strengthen health promotion and prevention, improve care coordination, and reduce avoidable healthcare costs and burden.

## Addressing systems challenges with health psychology

Healthcare systems across high-income countries face growing pressure from rising costs, workforce shortages, ageing populations, chronic disease burden, and increasing mental health needs (Deml et al., 2022; OECD/European Union, 2022; Patel et al., 2018). At the same time, many health outcomes depend on sustained behaviour change, self-management, and psychosocial resources. Despite growing interest in integrated and person-centred care, behavioural and psychosocial expertise remains insufficiently embedded within healthcare systems (Geese & Schmitt, 2023; Schmitz et al., 2017; World Health Assembly 69, 2016). These barriers highlight the need for more sustainable healthcare models that integrate the psychological dimension of care (Dekker et al., 2023; Thurston et al., 2023; World Health Organization Regional Office for Europe, 2024).

Health psychology is particularly well positioned to address system challenges through its focus on behavioural processes related to health, illness, and healthcare (Johnston, 1994). Grounded in biopsychosocial (Schramme, 2023) and salutogenic (Antonovsky, 1996) perspectives on health, the discipline develops targeted evidence-based approaches to health promotion, prevention, and integrated care. Growing evidence supports the cost-effectiveness of behavioural interventions across promotion and prevention (Le et al., 2021). Moreover, health psychology offers interprofessional interventions that can improve the effectiveness of specialised mental healthcare services (Colizzi et al., 2020). Several European healthcare systems have recently expanded first-line integrated care models, reflecting increased recognition of psychological professions in healthcare delivery (Darcis et al., 2022; Derksen, 2009; Infurchia & Lelievre, 2024; Liuzzi, 2020; Trautman & Beesdo-Baum, 2017). However, health psychology expertise is currently fragmented, locally implemented, and intervention-specific.

Health psychology operates within an interdisciplinary landscape and shares areas of research and practice with neighbouring fields. Its distinctive contribution lies in applying psychological science to behavioural and psychosocial processes across health, illness, and healthcare (Sutton et al., 2004). Clinical psychology and psychological medicine (i.e. in consultation-liaison psychiatry) have some overlap with health psychology in addressing psychological and psychiatric conditions in medical settings, but they focus on psychological illness rather than sustaining mental health. Behavioural medicine shares the behavioural focus with health psychology, but represents a broader interdisciplinary framework. Both health psychology and public health have a population-level perspective, but health psychology specialises in the mechanisms of individuals and groups within systems. Implementation science complements health psychology by supporting the translation, adaptation, and uptake of behavioural evidence into routine practice and policy (Presseau et al., 2022). These disciplinary boundaries are neither fixed nor mutually exclusive; health psychology therefore provides complementary expertise within interdisciplinary healthcare systems, although this overlap can also complicate the definition of professional roles and responsibilities.

In practice, the structural integration of health psychology remains uneven. In Switzerland, for example, financing for health psychology services remains unresolved, even though it is one of five recognised psychological professions in the law (PsyG, 2011) and postgraduate educational pathways are established. Similar barriers have been reported in the United Kingdom, including uncertainty regarding the organisational fit and professional role of health psychologists and difficulties in securing investment in discipline growth (Hart et al., 2023). More broadly, the development of psychologically informed healthcare requires attention to professional recognition, financing, and cross-disciplinary training (Dekker et al., 2023). When this expertise is not structurally integrated, healthcare systems may miss opportunities to translate behavioural evidence into routine practice and policy. This may leave health psychology know-how confined to isolated interventions or late referrals rather than embedded across prevention and care pathways (Dekker et al., 2023; Hart et al., 2023; Presseau et al., 2022). In lower-resource settings, where specialist workforce availability may be limited, integration may require different organisational models, including task-sharing and incorporation of health psychology expertise into primary care and community-based services (Le et al., 2022; Patel et al., 2018). Existing contributions have focused on specific domains such as behaviour change in healthcare quality or primary care integration (Doyle et al., 2023; Hilton, 2023), while broader integrated-care approaches remain isolated and insufficiently oriented toward population health and prevention (Noor et al., 2023). This clashes with the growing global need for integrated healthcare systems (World Health Assembly 69, 2016).

The present paper addresses this gap by proposing a systems-oriented framework for integrating health psychology across a continuum of health promotion and prevention. We primarily address public-health and healthcare-policy stakeholders, including decision-makers, healthcare leaders, and professionals involved in the organisation and development of healthcare systems. The paper aims to illustrate how health psychology can contribute to current system-level priorities and to identify opportunities for a more systematic integration into policy, prevention strategies, and care pathways. The system-oriented framework was developed through iterative conceptual discussion among the authors, members of the Swiss Society for Health Psychology. It is informed by established health psychology theories and selected literature from health psychology, behavioural medicine, public health, and integrated care. Supporting evidence was selected to substantiate and illustrate the framework, prioritising systematic reviews, large-scale studies, and authoritative policy sources where available. As no systematic literature search was undertaken, the cited evidence should be regarded as illustrative rather than exhaustive.

## Systems-oriented framework for integrating health psychology into sustainable healthcare

Behavioural and psychosocial processes lie at the core of health psychology and operate across these levels of the healthcare system. In health promotion and primary prevention, psychological science contributes to strengthening health literacy, resilience, healthy environments, and behaviour change. Secondary and tertiary prevention focus on coping, stress management, self-efficacy, adherence, and the reduction of the psychosocial consequences of illness. At the quaternary level, psychological expertise can help prevent over-medicalisation through appropriate care, communication, shared decision-making and care coordination. This framework further highlights that the organisation and financing of psychological interventions may differ across the prevention continuum. In line with the WHO distinction between health promotion and disease prevention, health promotion primarily focuses on strengthening psychosocial resources, adaptive capacities, and supportive environments rather than addressing pathology itself (Antonovsky, 1996; World Health Organization, 1986). Such interventions are therefore often supported through public health, educational, workplace, or community-based funding. By contrast, interventions targeting disease progression, coping, adherence, and clinical management increasingly fall within the remit of healthcare services and insurance coverage, particularly from secondary prevention onward, given their clinical and cost-effectiveness implications (World Health Organization/World Organization of Family Doctors, 2008) (See Figure 1).

**Figure 1.**
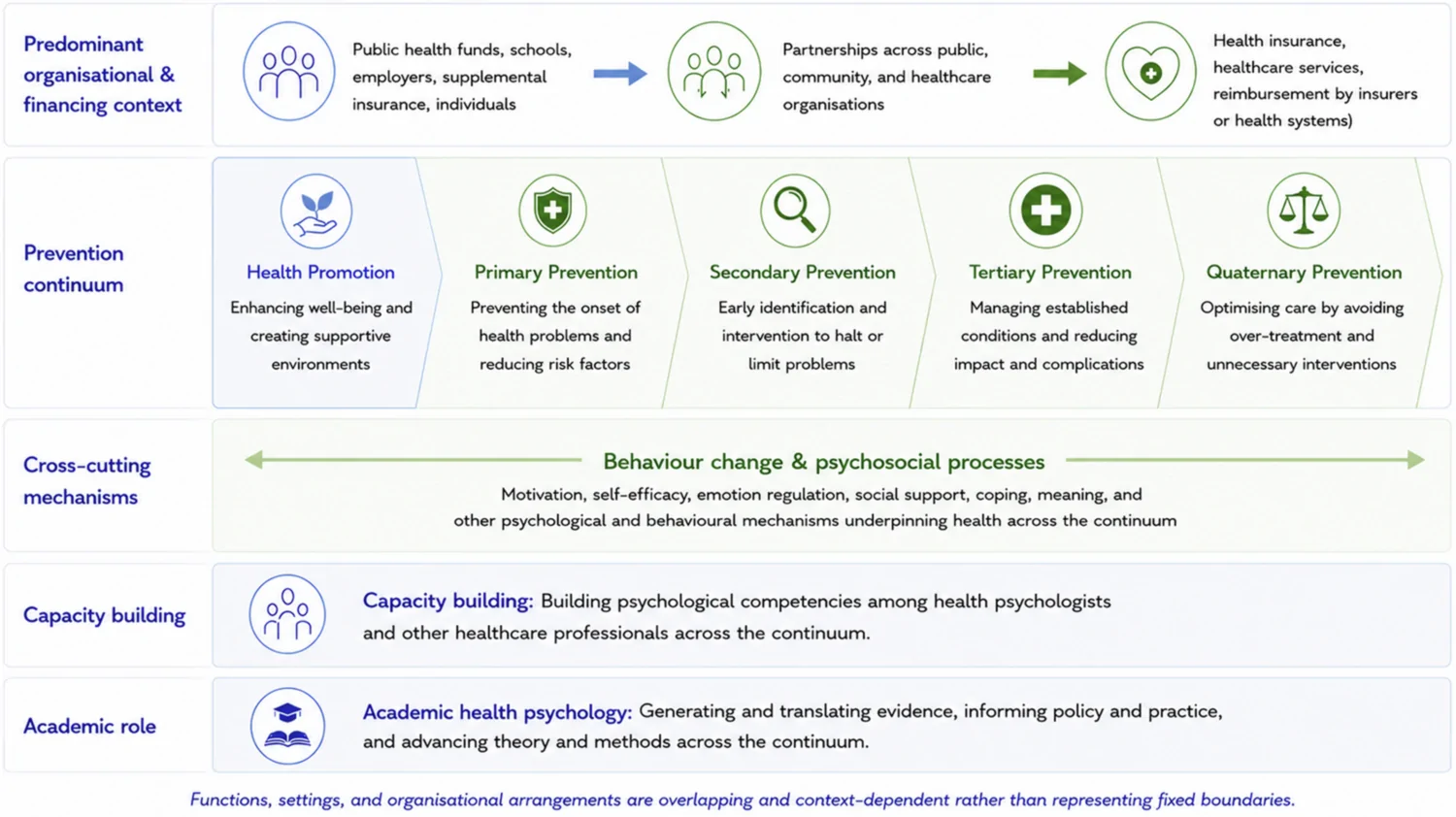
Health psychology as a continuum across prevention levels and organisational and financing contexts. The framework illustrates health psychology across the continuum from health promotion to quaternary prevention, highlighting cross-cutting behavioural and psychosocial processes and predominant organisational and financing contexts. Academic health psychology provides a foundation for evidence generation and translation across the continuum, while capacity building supports the integration of psychological competencies among health psychologists and other healthcare professionals. Prevention levels and organisational and financing arrangements are overlapping and context-dependent rather than representing fixed boundaries.

The framework conceptualises health psychology as a continuum across different organisational and governance levels. In practice, health psychology brings together professionals contributing to three interrelated domains: (1) research, education, and policy support; (2) public health promotion and prevention in schools, workplaces, and communities; and (3) clinical integration in hospitals and primary care. These domains should not be understood as isolated subfields, but as complementary components of a coordinated approach to healthcare. Each is detailed in the following sections (See Table 1).

**Table 1.** Executive summary of health psychology’s contribution to healthcare systems.

Subfield	Main focus	Key activities and settings	Contribution to healthcare systems
1. Academic Health Psychology	Research, education, and policy advice	Conducts research on health behaviour, self-management, and behaviour-change models Develops and translates evidence-based frameworks for prevention, communication, and implementation Provides academic and postgraduate training for psychologists and healthcare professionals Contributes behavioural expertise to policy and public health planning	Strengthens evidence-based policy and healthcare practice Supports development, implementation and evaluation of interventions Improves communication, adherence, and patient engagement across healthcare settings Promotes cost-effective and theory-informed prevention strategies
2. Public Health Promotion and Prevention	Health promotion and prevention in school, organisational, and community settings	Develops school-based health promotion and life-skills interventions Supports workplace health, safety, and psychosocial well-being Designs behaviourally informed prevention campaigns Advises educational, occupational, and public health stakeholders	Promotes healthy lifestyles and psychosocial well-being Strengthens resilience and health literacy Improves equitable health promotion through context-sensitive interventions Supports long-term prevention and population health
3. Clinical Health Psychology	Integration of behavioural and psychosocial care within healthcare systems	Provides behavioural assessment and intervention in hospitals and primary care Supports coping, adherence, self-management, health communication, and shared decision-making in chronic conditions Provides psychological consultation-liaison and contributes to psycho-oncology and primary care integrated care teams Implements integrated behavioural care models (e.g. PCBH) and digital interventions	Improves patient outcomes and care experience Enhances interprofessional collaboration, health communication and care coordination Reduces avoidable healthcare utilisation and and supports workforce well-being Supports sustainable, person-centred healthcare delivery

## Academic health psychology: bridging evidence and practice

Academic health psychology provides the evidence-generating and translational foundation for integrating psychological and behavioural knowledge into healthcare practice, policy, and system-level decision-making. The discipline generates evidence on health behaviour and related psychosocial processes. Complementing this research role, it provides graduate and postgraduate academic education for psychologists. It also contributes to capacity building across healthcare systems by equipping healthcare professionals with competencies to understand patient behaviour, communicate effectively, and support motivation, shared decision-making, and treatment adherence.

A major contribution of the discipline has been the development of evidence-based theoretical models such as the Health Belief Model (Rosenstock, 1977), the Theory of Planned Behavior (Ajzen, 1991), the Health Action Process Approach (HAPA model) (Schwarzer, 2008), and more recent implementation-oriented frameworks such as the COM-B model of behaviour change (Michie et al., 2011). These models identify psychosocial determinants of behaviour and provide structured approaches for designing, implementing, and evaluating health interventions across individual, professional, organisational, and population levels. Meta-analytic evidence supports the utility of such frameworks in predicting and changing health behaviours across contexts (Xu et al., 2026; Zhang et al., 2019). Importantly, health psychology theories and methods are increasingly translated into implementation strategies targeting behaviours at different levels of healthcare systems (Presseau et al., 2022). For example, a large theory-based trial involving healthcare professionals showed that implementation-intention approaches increased the delivery of behaviour-change interventions during routine clinical consultations (Keyworth et al., 2026). At the system level, the English National Health Service Diabetes Prevention Programme provides an example of population-scale implementation, with behaviour-change theory and techniques explicitly incorporated into its service specification (Hawkes et al., 2021) and routine service data demonstrating improvements in weight and HbA1c following nationwide implementation (Valabhji et al., 2020). Evidence from low- and middle-income countries further indicates that behaviour-change interventions can improve dietary adherence and related health outcomes across diverse settings, with specific techniques such as action planning and self-monitoring associated with greater effects (Caperon et al., 2018). The relevance of behaviour-change theories at the population level was also highlighted during the COVID-19 pandemic, particularly for understanding and promoting adherence to preventive health behaviours across countries and diverse populations (Baretta et al., 2025; Petrocchi et al., 2021; Schumpe et al., 2022). Together, these examples illustrate how behavioural theory can inform improvements ranging from healthcare-professional practice to population-scale service delivery.

Nevertheless, evidence-based interventions do not automatically become routine practice or policy, with barriers operating at professional, organisational, and system levels. Closer integration between health psychology and implementation science can help identify these barriers and support the adaptation, uptake, and evaluation of evidence-based approaches in real-world settings (Presseau et al., 2022). For healthcare systems, this calls for a translational approach that embeds health psychology evidence into policy and practice.

## Public health promotion and prevention: embedding behavioural expertise in everyday settings

Health promotion and prevention go beyond healthcare settings (Naidoo & Wills, 2016). While primary prevention serves to intervene at an early stage to prevent diseases or problematic behaviours or mitigate their consequences, health promotion aims to empower people to build physical, mental, and social resources so that they can shape and improve their health in a self-determined manner. A distinction is made between who or what is specifically addressed: at the individual level, health literacy and psychosocial competencies are strengthened, and at the environmental level, living environments are designed to promote health (Dadaczynski & Paulus, 2018). From a systems perspective, integrating this expertise into schools, workplaces, and communities provides an opportunity to address health determinants upstream, before needs translate into greater demand for healthcare.

### Health promotion and prevention in schools

Schools provide an important setting for population-level health promotion because they can reach young people during formative developmental periods. Health psychology can inform whole-school and targeted approaches that strengthen health literacy, life skills, self-regulation, informed decision-making, and resilience (Achermann Fawcett et al., 2018; UNICEF, 2019). Evidence from the WHO Health Promoting Schools framework supports the potential of such settings-based approaches. A Cochrane review of cluster-randomised trials found beneficial effects across several health behaviours and outcomes (Langford et al., 2015), while a recent systematic review and meta-analysis of whole-school interventions reported reductions in smoking, cyberbullying, and cyber-aggression among adolescents, while highlighting implementation and contextual adaptation as important determinants of effectiveness (Lekamge et al., 2025). Beyond targeting individual behaviours, health psychology can contribute to shaping school environments that support well-being and healthy development, linking individual competencies with the social conditions in which behaviours occur (Kirchhoff & Keller, 2021). For public health systems, embedding such approaches within educational structures offers a scalable means of population-level prevention.

### Health promotion and prevention in organisations

Organisations represent another important setting for health promotion and prevention, as working conditions can both support and threaten physical and psychological health. Occupational health psychology contributes by addressing the interaction between working conditions, organisational processes, and individual behaviour to promote worker health, safety, and well-being (Gonçalves et al., 2022). Evidence-based approaches can complement structural and regulatory measures by targeting determinants of health and safety behaviours.

Their application in real-world settings is illustrated by the Swiss National Accident Insurance Fund (SUVA) UV-protection campaign, which applied behaviour-change principles to address psychosocial determinants of sun-protective behaviours among outdoor workers (Büchel et al., 2018). Although behaviourally informed campaigns require investment in understanding key determinants and implementation contexts, this can increase their effectiveness in achieving sustained change (Ramezani et al., 2022).

Community and organisational settings therefore provide an important bridge between behavioural evidence and real-world prevention practice. Embedding health psychology expertise within these settings can strengthen workplace safety, disease prevention, and long-term population health.

## Clinical integration: embedding behavioural and psychosocial expertise in healthcare

Psychologically integrated care aligns with the Quadruple Aim of improving patient experience, population health, healthcare sustainability, and provider well-being (Bodenheimer & Sinsky, 2014). Hospitals and primary care settings provide key opportunities to structurally embed health psychology expertise within interprofessional care pathways, supporting patient communication, self-management, treatment adherence, shared decision-making and care coordination (World Health Organization/World Organization of Family Doctors, 2008). Rather than substituting for specialised mental healthcare, this integration addresses psychological dimensions of health as part of routine care and facilitates timely referral when specialised treatment is required.

### Addressing behavioural health in hospital settings

Mental health conditions frequently co-occur with physical illnesses and may require specialised psychiatric or psychotherapeutic care. However, hospitalised patients also present psychological needs related to illness, treatment, and adaptation that do not necessarily require specialised mental healthcare. Such needs may benefit from low-threshold, early interventions delivered by health psychologists integrated with other healthcare specialists (physicians, nurses, physiotherapists, nutritionists, social workers). For example, in acute settings, psychological interventions integrated during or following intensive care have shown potential benefits for post-traumatic stress, depression, and quality of life following critical illness (Cesari et al., 2026). Meta-analytic evidence further indicates that family-support interventions promoting communication and shared decision-making in intensive care can reduce ICU and hospital length of stay, illustrating potential system-level benefits of integrated care (Lee et al., 2019). Furthermore, health psychology may also contribute to patient-centered communication in emotionally challenging situations, particularly around prognosis, hope, and complex treatment decisions (Grignoli et al., 2021; Qama et al., 2021). Together, these examples illustrate how psychological expertise can complement disease-focused hospital care. More broadly, evidence from psychiatric and psychosomatic consultation-liaison services supports the benefits of integrated approaches for psychological outcomes among medically ill hospital patients (Stein et al., 2020).

### Behavioural interventions in primary care and chronic disease management

Beyond acute care, health psychologists can contribute to the management of chronic conditions by addressing illness-related behaviours. Chronic diseases require significant lifestyle changes, which can be difficult for patients to sustain and for physicians to treat without interprofessional integration (Chiolero et al., 2020). Health psychologists support lifestyle changes through evidence-based approaches, including use of coping assessment and motivational interviewing techniques (Friedman, 2011). Diabetes, for example, has benefited from behavioural interventions supporting patient self-care (Chew et al., 2017). Pain management represents another core intervention in health psychology (Hoffman et al., 2007), with evidence supporting scalable, ultra-brief (single-session) interventions (Darnall et al., 2021). Psycho-oncology similarly applies health psychology principles to address psychosocial needs alongside cancer care (Holland et al., 2021), including fear of recurrence for which psychological interventions have demonstrated benefit (Sanjida et al., 2018). Economic evidence further supports the cost-effectiveness of psychological interventions across physical health conditions (Nicklas et al., 2022).

While these examples illustrate the contribution of health psychology within hospital and specialist care, primary care represents a key setting for the long-term management of chronic health conditions and therefore provides a particularly relevant context for systematic integration. Integrated models such as Primary Care Behavioural Health (PCBH) have demonstrated clinical efficacy across different levels of symptom severity (Bryan et al., 2012; Cigrang et al., 2007). PCBH can improve access to and engagement with behavioural health care, while real-world evidence also indicates potential reductions in healthcare utilisation, including emergency department and primary care visits (Maeng et al., 2022; Possemato et al., 2018). Embedding psychologists within primary care may also reduce the time burden on primary care providers while generating cost savings (Gouge et al., 2016; Ross et al., 2019). Such integrated approaches may become increasingly important given the rising prevalence of chronic diseases and the often-unmet psychosocial needs (Bergerot et al., 2024; Berkel et al., 2019; Essue et al., 2020). Together, these findings suggest that integrated behavioural care models may contribute to more sustainable healthcare delivery by improving efficiency, reducing avoidable healthcare utilisation, and alleviating pressures on the healthcare workforce.

## Limitations and future perspectives

As a conceptual and systems-oriented perspective, the present article synthesises selected evidence to illustrate the potential contribution of health psychology across healthcare systems. The examples are illustrative rather than exhaustive and may not fully capture variations across healthcare systems and sociocultural contexts. The proposed framework should therefore be understood as identifying adaptable functions of health psychology rather than prescribing a uniform model of integration.

For public health and healthcare decision-makers, translating these functions into sustainable practice requires attention to several system-level conditions, including governance, financing and reimbursement mechanisms, workforce capacity, professional roles, interprofessional collaboration, and organisational readiness for change. Two challenges may be particularly relevant to integrated models. First, integration can blur established professional boundaries and create uncertainty regarding roles, responsibilities, and accountability; conversely, clear role definition and shared responsibilities can facilitate interprofessional care (Best & Williams, 2019). This is particularly relevant where health psychology overlaps with neighbouring disciplines, requiring roles to be defined according to local competencies and regulations. Second, reorganising established care pathways may generate resistance when new roles challenge professional identities, routines, or responsibilities (Best & Williams, 2019; Schubert et al., 2023). Such resistance may reflect legitimate concerns about professional identity, accountability, autonomy, and role redistribution rather than opposition to integration itself. Implementation should therefore build on existing healthcare and public-health structures and adapt to local contexts. Particular attention is required to equitable reach, as interventions embedded in schools, workplaces, or healthcare services may not adequately reach populations with limited access to these settings, including young people outside formal education, people outside formal employment, and underserved migrant communities (Kennedy et al., 2026).

Future research should build on the proposed framework to develop and evaluate models of coordinated health psychology integration at scale, examining their long-term impact and the conditions that support successful implementation across healthcare systems. This should include real-world data on patient and population health outcomes, healthcare utilisation, prevention effectiveness, workforce well-being, equity, and cost-effectiveness, as well as comparative evaluation of how different organisational, financing, and regulatory contexts influence implementation and sustainability.

## Conclusions: building bridges for more sustainable healthcare systems

Integrating behavioural and psychosocial expertise into healthcare systems can contribute to person-centred and cost-effective care. Through research and policy advice, health psychologists can provide evidence to support systemic change. Public health and prevention initiatives empower individuals and communities to improve their well-being, while clinical integration enhances care by addressing the behavioural dimensions of health. To fully realise this potential, psychological expertise should be structurally embedded within healthcare governance, training, prevention strategies, and clinical pathways. This requires clearer professional roles, sustainable financing and implementation models, and stronger collaboration across medical, nursing, public health, educational, and policy sectors.

Moving beyond isolated behavioural interventions toward coordinated and context-sensitive integration of behavioural and psychosocial expertise may support the development of more sustainable healthcare systems.

## Data Availability

Data sharing is not applicable to this article as no new data were created or analysed in this study.
